# RELATIONSHIP BETWEEN SELF-COMPASSION, ASSERTIVENESS AT WORK AND JOB SATISFACTION AMONG TEACHERS

**DOI:** 10.13075/ijomeh.1896.02574

**Published:** 2025

**Authors:** Iga Komorowska, Dagna Kocur, Katarzyna Ślebarska, Justyna Lipka, Aleksandra Żenda

**Affiliations:** University of Silesia in Katowice, Institute of Psychology, Katowice, Poland

**Keywords:** job satisfaction, well-being, teachers, self-compassion, assertiveness, educators

## Abstract

**Objectives:**

In the study, the authors aimed to explore the relationship between self-compassion, assertiveness and job satisfaction among teachers. Specifically, they examined whether assertiveness mediated the relationship between self-compassion and job satisfaction, filling a gap in the existing research on teachers' well-being.

**Material and Methods:**

A total of 208 teachers (192 women, 16 men) aged 24–64 years, with an average teaching experience of 21 years, participated in the study. The participants were recruited using snowball sampling. Self-compassion was measured with the *Self-Compassion Scale* (SCS), assertiveness with the *Teacher Assertiveness Questionnaire*, and job satisfaction with the *Job Satisfaction Scale*. Statistical analyses included descriptive statistics, Spearman's correlation and mediation analysis using Process tool (model 4).

**Results:**

Self-compassion was positively correlated with both assertiveness (r = 0.21, p < 0.01) and job satisfaction (r = 0.18, p < 0.05). Assertiveness was also positively related to job satisfaction (r = 0.21, p < 0.01). Mediation analysis demonstrated a total mediating effect of assertiveness in the relationship between self-compassion and job satisfaction, with the model explaining 8.3% of the variance in job satisfaction.

**Conclusions:**

The findings suggest that self-compassion promotes teachers' assertiveness, which in turn increases job satisfaction. This highlights the importance of supporting self-compassion and assertiveness as resources protecting against occupational stress and burnout. However, the cross-sectional nature of the study limits causal inference, so future research should consider longitudinal models and different educational contexts. These findings provide practical insights for the design of interventions aimed at promoting teacher well-being.

## Highlights

- Self-compassion correlates positively with job satisfaction among teachers.
- Assertiveness mediates the relationship between self-compassion and job satisfaction.
- Higher self-compassion supports assertiveness in workplace interactions.
- Findings highlight the potential of self-compassion training for teacher well-being.

## INTRODUCTION

Over the past 2 decades, the phenomenon of self-compassion has received much attention in the field of social and clinical psychology. Self-compassion, conceptualized by Neff [1] as an emotionally positive self-attitude, had already been present in Buddhist thought before it was introduced in Western psychology [2]. According to Neff [3], self-compassion “represents compassion turned inward and refers to how we relate to ourselves in instances of perceived failure, inadequacy, or personal suffering” (p. 265). The development of research on self-compassion has been linked to the search for alternatives to self-esteem, which entails the need to make social comparisons and is sometimes related to the quest for superiority. Unlike self-pity, self-compassion includes a sense of unity with others who are suffering [4]. Self-compassion contains 3 elements: self-kindness (vs. self-judgment), common humanity (perceiving suffering as an experience shared by humanity as opposed to isolation) and mindfulness, contrasted with overidentification [4]. In Ewert et al.'s [5] meta-analysis, self-compassion was examined as a personal resource and revealed to be associated with adaptive coping. Other meta-analyses showed an association between self-compassion and psychological well-being, including its psychological and cognitive aspects [6], positive relationships between self-compassion, physical health, and health behavior [7]. Interestingly, self-compassion can be enhanced through psychological interventions and training programs [8,9]. In summary, self-compassion is a psychological construct with well-documented potential to support individual well-being, which makes its relevance in the professional context – especially among occupational groups such as teachers, who are particularly vulnerable to stress and burnout – worthy of in-depth investigation.

Recently, there has been a growing interest in self-compassion among teachers and educators. The analysis of the links between self-compassion and elements of work well-being appears meaningful given the systemic challenges and work-related problems faced by teachers, as well as the changing status of the teaching profession in many countries, contrasted with beneficial outcomes originating from self-compassion [10]. Previous analyses include concerned, among other things, associations between self-compassion and educator stress [11], the moderating effect of self-compassion in the relationship between job stress and job-related affective well-being [12], and self-compassion as a predictor of teacher resilience [13]. A challenging area in the research on self-compassion is the latter's associations with well-being related to professional life. A systematic review by Kotera and Gordon [14] revealed that self-compassion training can enhance work-related well-being. Selected papers include the research of Maratos et al. [15] and Beshai et al. [16] conducted among teachers/educators. An interesting area of inquiry regarding self-compassion among teachers concerns the association between self-compassion and job satisfaction. Previous work addressed and revealed this relationship among white-collar workers [17], nurses [18], and business leaders, among whom self-compassion served as a mediator between communication competence and job satisfaction [19]. Moreover, in Babenko et al. [20] study, self-compassion was positively associated with professional life satisfaction. Therefore, the authors considered it important to further investigate the relationship between self-compassion and job satisfaction among teachers, and to examine potential mediators of this relationship. One such mediator may be communication skills such as assertiveness, as they have been shown to be associated with both self-compassion and job satisfaction [19].

An intriguing yet underinvestigated research direction concerns the relationship between self-compassion and assertiveness, defined as “the ability of an individual to express their feelings or thoughts without violating others' rights” [21] (p. 1). Drawing on Poprawa's definition of assertiveness as “a personality disposition for a specific way of self-expression of one's beliefs and feelings and coping with the demands of life relationships mainly concerning interpersonal contacts'” [22] (p. 114), Zubrzycka-Maciąg and Kirenko [23] distinguished 4 areas in which teachers' assertiveness can be manifested: with students, with students' parents, with other teachers and with the principal/supervisor, concerning aspects such as defending one's rights, accepting positive and negative evaluations, making requests, expressing feelings, giving opinions and respecting others' boundaries. Importantly, the researchers show that according to Poprawa's conceptualization, one of the components of assertiveness are beliefs “about one's own qualities and self-worth”, related to “positive self-esteem and a sense of self-confidence and self-acceptance” [23] (p. 14). Only a few studies have been published on self-compassion links to assertiveness, including Ghafarian and Khayatan's research [24] that revealed an impact of compassion-focused therapy on assertiveness, as well as Akin's [25] study in which self-compassion was revealed to be negatively correlated with submissive behavior. None of the aforementioned studies concern assertiveness in work among teachers and educators. Given the effectiveness of self-compassion training and the possibility of developing it, as well as the importance of assertiveness as a negative predictor of emotional exhaustion among teachers [26], filling the knowledge gap seems vital. Therefore, this study aims to examine the relationship between self-compassion, job satisfaction and assertiveness in work among teachers.

### The present study

A review of the existing literature provides ample evidence of the beneficial effects of higher levels of trait self-compassion as well as of interventions developing self-compassion for functioning in interpersonal relationships in the professional sphere [14,19,27]. Many studies also confirm positive associations between self-compassion and job satisfaction [28,29], also among teachers [30,31]. The authors thus hypothesize positive correlations between self-compassion and job satisfaction among teachers (H1).

Research to date has not confirmed the relationship between self-compassion and the level of assertiveness. However, several studies can be indicated that may point to an indirect relationship between these variables. Akin [25] notes that submissive behaviors correlate negatively with 3 elements of self-compassion: self-kindness, common humanity, and mindfulness. In a meta-analysis, Ewert et al. [5] highlighted the positive relationship between self-compassion and adaptive coping methods. In addition, a study using compassion focused therapy showed a significant increase in assertiveness after only 8 therapy sessions, but this result was not maintained in the follow-up [24]. The authors thus hypothesize positive correlations between self-compassion and assertiveness at work among teachers (H2).

Research confirms relationships between assertiveness and job satisfaction ratings. For example, more assertive respondents in groups of nurses [32] and physicians [33] were found to rate their job satisfaction higher. An analysis concerning prospective teachers proved that assertiveness might influence perceived job satisfaction due to the association of assertiveness with aspects such as well-being and self-control [34]. Thus, the authors hypothesize positive correlations between assertiveness and job satisfaction among teachers (H3).

**Figure 1:**
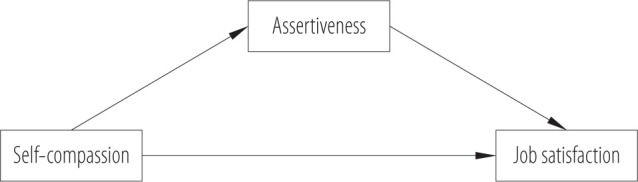
Research model of the relationship between self-compassion, assertiveness and job satisfaction among teachers (N = 208), January–March 2024, Poland

It is important to investigate the mediating effect of assertiveness in the relationship between self-compassion and job satisfaction, as assertiveness – the ability to express one's needs and emotions while respecting others – plays a crucial role in teachers' daily functioning [35]. According to self-compassion theory [4], self-compassion fosters emotional regulation and psychological resilience, which can facilitate assertive communication in the workplace. Assertiveness, in turn, supports job satisfaction by enabling teachers to maintain boundaries, manage workload, and cope with interpersonal demands more effectively. Moreover, research shows that self-compassion is a strong predictor of job satisfaction beyond factors such as affective commitment and job tenure [36]. Mediation models suggest that self-compassion may enhance job satisfaction indirectly by reducing burnout [37]. Since assertiveness is negatively associated with burnout [38], it may serve as a protective mechanism through which self-compassion contributes to professional well-being. Moreover, self-compassion promotes greater authenticity and the initiative to make needed changes [39,40]. According to Neff [4], self-compassion “can also take a fierce, powerful, agentic form, especially when it is aimed at self-protection, meeting the important needs or motivating change.” Thus, self-compassion can be understood as a more profound personal resource that facilitates boundary-setting. As pointed out by Austin et al. [41], “These fierce aspects of self-compassion, such as encouraging growth and drawing boundaries, facilitate getting back to a meaningful life after dealing with adversity.” Among the possible actions (“committed action steps”), researchers mentioned developing assertiveness skills. Also in the study by Ghafarian and Khayatan [42], compassion-focused therapy significantly affected assertiveness. Thus, the authors hypothesize that teachers' assertiveness at work mediates the relationship between self-compassion and job satisfaction (H4) (Figure 1).

## MATERIAL AND METHODS

### Participants

The study group consisted of 208 respondents, including 192 (92.31%) women and 16 men (7.69%), with an age range of 24–64 years. The gender percentage distribution was not even, which reflects the employment structure of teachers in Poland in terms of gender. According to data from the Ministry of National Education, 84.2% of teachers in Poland are women [43]. Half of the respondents were aged ≤48 years [median (Me) = 48 years, mean (M) ± standard deviation (SD) 47±8.88 years]. All of the respondents had a higher education degree in teaching. The length of service of the respondents was M±SD 21±10.31 years. Detailed sociodemographic data are presented in Table 1.

**Table 1:** Sociodemographic characteristics of the study sample, January–March 2024, Poland

Variable	Participants (N = 208)	
	n	%
Gender		
female	192	92.31
male	16	7.69
Education		
higher	208	100
Place of residence		
village	75	36.06
city		
10–100 000 inhabitants	49	23.56
100 000–500 000 inhabitants	72	34.62
>500 000 inhabitants	12	5.77
Subjects taught		
early childhood education	54	25.96
humanities	71	34.13
science	49	23.56
arts and PE	34	16.35
Facilities		
pre-school	28	9.9
primary school		
grades 1–3	92	32.4
grades 4–8	136	47.9
vocational school	1	0.4
technical school	8	2.8
secondary school	11	3.9
other	8	2.8

### Procedure

All data were collected using the snowball sampling method among Polish teachers. No incentive was offered for participation in the study. The participants were volunteers and were informed about the purpose of the study. Ethical approval for the study was granted by the Institutional Review Board No. KEUS358/03.2023. The data were collected in January–March 2024 in Poland. The study targeted adults with pedagogical education who were professionally active teachers at the time of participation. The survey was administered via the LimeSurvey platform [44] and in paper form. Invitations were distributed to educational institutions and teacher training centers in the Silesian Voivodeship via email, as well as through teacher-specific online forums. Paper-based participation was offered through selected institutions in the same region. The inclusion criterion was being a professionally active teacher with pedagogical education. The only exclusion criterion was incomplete responses to the questionnaire; 34 responses were excluded on this basis.

### Measures

Self-compassion was measured using the 26-item *Self-Compassion Scale* [45]. This tool consists of 6 subscales referring to the components of the studied variable: self-kindness, self-judgment, common humanity, isolation, mindfulness, and over-identification. Each item is measured using a scale from 1 (almost never) to 5 (almost always). In this study, the authors decided to analyze only the overall result. The reliability of the indicated tool was satisfactory in the research (Cronbach's α = 0.93).

The *Teacher Assertiveness Questionnaire* (*Kwestionariusz Asertywności Nauczycieli* – KAN) (area A) by Zubrzycka-Maciąg and Kirenko [24] was used to measure the teacher assertiveness variable. This questionnaire consists of 7 subscales – defending one's rights, accepting positive and negative evaluations, expressing positive and negative evaluations, expressing requests, expressing feelings, expressing opinions, and respecting others' boundaries. The subscales add up to an overall assertiveness score. The individual items are rated using a 5-point scale from 1 (completely false) to 5 (completely true). The reliability of the tool was satisfactory in the study (Cronbach's α = 0.84).

To study job satisfaction among teachers in the research the Job Satisfaction Scale (*Skala Satysfakcji z Pracy* – SSP) [46] was used. The tool consists of 5 items rated on a 7-point scale from 1 (strongly disagree) to 7 (strongly agree). The reliability of the tool was satisfactory in the study (Cronbach's α = 0.79).

### Data analysis

We used SPSS v. 29 for all statistical analyses. A correlation analysis was carried out using Pearson's r. The authors then conducted a mediation analysis using the Process v. 4.2 beta plugin by Andrew F. Hayes [47] in IBM SPSS (v. 29). The authors tested a mediation model (model 4) in which self-compassion is a predictor of job satisfaction through teacher assertiveness (mediator). The significance of the indirect effect in the mediation analyses was determined by examining whether 95% confidence intervals (CI) do not contain 0 values. In line with the recommendation of Abrar et al. [48], the authors presented standardized regression coefficients, and the size of the mediation effect was assessed based on the fully standardized indirect effect (abcs) [49]. The authors assumed that an effect size of 0.01 would indicate a small mediation, 0.09 a medium mediation, and 0.25 a large mediation [50]. Results were considered statistically significant if p < 0.05.

## RESULTS

The authors began the analyses by calculating the descriptive statistics and correlations between the study variables. The distribution of the variable self-compassion was close to normal (W = 0.99, p = 0.05), while the distributions of assertiveness and job satisfaction deviated from normality (p < 0.001). However, when analyzing the results of skewness and kurtosis, the distribution of job satisfaction was considered to be close to normal (Sk = –0.67, Κ = 0.74), and assertiveness turned out to have a left-skewed and leptokurtic distribution (As = –1.51, K = 3.39), but was still considered to be symmetrical [51]. We observed significant positive correlations (Table 2) between self-compassion on the one hand, and assertiveness and job satisfaction on the other hand. Assertiveness also correlated positively with job satisfaction.

**Table 2:** Descriptive statistics and results of the analysis of Spearman's correlation between the variables (N = 208), January–March 2024, Poland

Variable	M±SD	Spearman's ρ			
		1	2	3	4
1. Age	47.038±8.881	–			
2. Job seniority	21.274±10.307	0.918***	–		
3. Self-compassion	3.197±0.682	–0.002	–0.009	–	
4. Assertiveness	4.259±0.471	0.147*	0.172*	0.214**	–
5. Job satisfaction	4.716±1.129	0.022	0.064	0.175*	0.213**

Spearman's ρ coefficient was tested.

* p < 0.05;

** p < 0.01;

*** p < 0.001.

**Figure 2:**
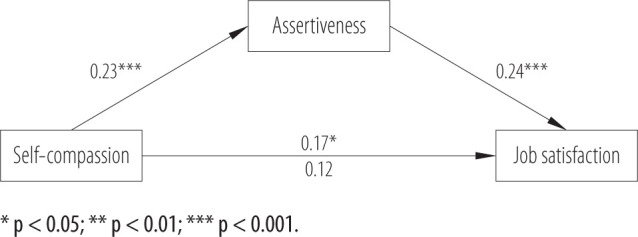
Standardized results of the mediation model of the relationship between self-compassion, assertiveness and job satisfaction among teachers (N = 208), January–March 2024, Poland

In the next step, a mediation analysis was carried out using Process v. 4.2 (model 4) [47]. The mediation model F(2, 205) = 9.33, p < 0.001 explained 8.3% of the variance. The results are presented in Figure 2.

According to the above, self-compassion positively correlates with assertiveness (β = 0.23, p < 0.001, and assertiveness with job satisfaction (β = 0.24, p < 0.001). The total effect of self-compassion on job satisfaction was statistically significant (β = 0.17, p = 0.01), while when assertiveness was included in this model, the direct effect was β = 0.12, p = 0.10, which indicates total mediation. Based on a completely standardized indirect effect the mediating effect has been shown to be small but statistically significant (abcs = 0.05, 95% CI: 0.02–0.10) [49,50].

## DISCUSSION

In the study, the authors aimed to examine the relationship between teachers' perceived self-compassion, their assertiveness at work, and their job satisfaction. An analysis of the results confirmed a significant positive relationship between self-compassion and job satisfaction ratings among teachers (H1). Previous results confirm this relationship [19,29–31]. The authors can therefore conclude that teachers characterized by being self-understanding and self-supportive, and those who realize the universal nature of suffering and failure, and do not separate themselves from others in such feelings, have a higher conscious rating of their work – of its conditions and of their achievements so far. It is interesting to expand on this phenomenon, especially in this occupational group, as studies have confirmed the association between practicing self-compassion and higher job satisfaction ratings [32,52]. Reduced job satisfaction can be related not only to one's own experiences – lack of meaning, frustration, fatigue or anxiety [53,54], but can also negatively affect work outcomes and relationships with students and parents [55]. The effects of interventions related to developing self-compassion on stress reduction [56,57] may be relevant from the point of view of teachers, as most of them experience exhaustion, stress and negative emotions in their work [58]. A good working relationship with students and parents was identified as one of the factors contributing to greater job satisfaction [59]. Studies also confirmed that higher mindfulness (which is a component of self-compassion) was associated with better relationships with students – there was less conflict and greater closeness [60]. The authors can therefore assume that developing self-compassion may be associated with more valuable interactions with students and with lower stress in teachers, which may in turn translate into an association with higher ratings of job satisfaction among the latter.

It is worth noting, however, that in the study, the correlation between self-compassion and job satisfaction was relatively low (r = 0.18), especially when compared to findings such as those by Abacı and Arda [17], where the strength of this relationship was reported as r = 0.45. Not all studies confirm a significant correlation between self-compassion and job satisfaction [61], nor do they consistently demonstrate an increase in job satisfaction following effective self-compassion interventions [62]. This discrepancy may be partly due to the use of different measurement tools. Therefore, this area requires further investigation, particularly studies that explore additional mediators or contextual factors that may influence these relationships.

The study also validated H2, assuming a link between higher self-compassion and higher assertiveness in teachers' work. Teachers still capable of being kind and understanding towards themselves in the face of difficulties and consciously accepting their thoughts and feelings while maintaining perspective are characterized by higher levels of assertiveness in their work. Those, on the other hand, who do not feel the universality of suffering and failure, over-identify with negative emotions and thoughts, and tend to be harshly judgmental and critical of themselves and of their own actions, may be less assertive in the performance of their job-related duties.

The authors' findings correspond with studies on the impact of self-compassion training on increasing assertiveness [22,25]. Well-developed self-compassion is linked to the ability to resolve conflicts in a productive and healthy manner [63]. Assertiveness is recognized as a supportive element in stressful situations and in dealing with criticism, which at the same time helps to build good relationships with students. In the same way, developing self-compassion may contribute to stress reduction. The associations between higher self-compassion and higher assertiveness in teachers' work thus appear to be complementary, as both skills can constitute protective factors against occupational burnout (with symptoms such as stress, negative emotions, and exhaustion). The link between higher mindfulness (a self-compassion element) and lower levels of burnout was confirmed in the research by Szoke et al. [64], while the protective effect of assertiveness was identified in a study by Jarmużek [65]. The authors can conclude that the link between higher self-compassion and higher assertiveness at work is an important trait of teachers due to the potential relations with more valuable contacts and buffering of negative emotions related to teachers' work.

The study confirmed the hypothesized relationship between experiencing higher assertiveness in teachers' work and higher job satisfaction ratings (H3). These findings seem to have been confirmed also in studies conducted among nursing personnel [66] and physicians [34]. The authors can therefore conclude that teachers characterized by higher assertiveness rate their work higher. What is particularly significant is that assertiveness mediates the relationship between self-compassion and job satisfaction among teachers (H4). Significantly, according to Neff [4], self-compassion “can also take a fierce, powerful, agentic form, especially when it is aimed at self-protection, meeting the important needs or motivating change.” This is an aspect of self-compassion usually associated with assertiveness, in this case related to compassionate self-protection. Teacher assertiveness seems to be the resource that explains the relationship between self-compassion and job satisfaction. High self-compassion means self-kindness in difficult work situations, while assertiveness supports actions aimed at protecting oneself, for example by setting boundaries or achieving personal goals [24]. All these relationships can lead to a conclusion that a teacher with high self-compassion is also an assertive teacher with a high rating of their work and positive thoughts about it. The authors cannot infer the direction of these relationships here, but many studies have shown that developing self-compassion has a positive impact on overall mental health [67], redu ces stress levels [56], and contributes to productive conflict resolution [63], which can be very helpful in coping as teachers. Given the problems that are reported by this occupational group (fatigue, negative affect, stress) and the impact of self-compassion on mental health, it seems appropriate to consider complementing and developing teachers' self-compassion skills.

Although the obtained results are significant, it is worth noting that their direct effect is only 0.18, indicating a relatively weak strength of this relationship. In the future, it would be valuable to explore additional mediators of this relationship that could complement the model and strengthen the explanation of this phenomenon.

### Limitations

The study has several important limitations. The gender distribution in the sample is unequal, with a prevalence of women (92.31%). This imbalance reflects the gender distribution in the population, but makes it impossible to capture potential gender differences in self-compassion, assertiveness, and job satisfaction. In addition, the use of snowball sampling may have led to the recruitment of participants with similar demographic or occupational characteristics, which also affects the representativeness of the results. The study was based solely on self-report questionnaires, and its cross-sectional nature makes it impossible to establish clear causal relationships between self-compassion, assertiveness, and job satisfaction. The lack of measurement of potential confounding variables, such as the level of occupational stress or social support, further limits the full understanding of these relationships, so it is worth considering a greater number of contextual factors and confounding variables in future studies, and furthermore it is worth ensuring a better diversity of respondents in the teachers' working environment, in terms of the school type and educational level of students. Valuable results would be provided by a study using interventions developing self-compassion, where it would be checked after a prolonged period of time how the development of self-compassion has affected assertiveness and job satisfaction.

## CONCLUSIONS

This study highlights the importance of self-compassion as a resource boosting both assertiveness and job satisfaction among teachers. The findings confirm that a higher level of self-compassion is associated with greater assertiveness in the workplace, as well as higher job satisfaction. Furthermore, the authors have demonstrated the mediating role of assertiveness in the relationship between self-compassion and job satisfaction, suggesting that self-compassion supports teachers in cultivating assertive behaviors, which in turn positively influence how they evaluate their job.

Given the challenges teachers face in their professional environment, supporting self-compassion and assertiveness can be a practical and effective strategy to improve job satisfaction and reduce professional stress. These observations highlight the need for further research into interventions aimed at developing self-compassion and assertiveness, as well as exploring their longterm impact on teachers' resilience and occupational fulfilment.
